# Prokaryote-Eukaryote Symbiosis to Produce RNA-Based Therapeutics

**DOI:** 10.3389/fgene.2020.583464

**Published:** 2020-10-16

**Authors:** Behdad Tahayori

**Affiliations:** Department of Physical Therapy, University of Saint Augustine for Health Sciences, Miami, FL, United States

**Keywords:** COVID-19, *Dictyostelium discoideum*, symbiosis, ncRNA, RNA based therapeutics

## Introduction

Engineering genetic material for treatment of disease is a promising method which has been used for the treatment of different human pathologies for several decades (Anderson, 1984). Theoretically, RNA-based therapeutics can be used for a wide range of diseases from Alzheimer to HIV and Influenza viruses. However, several barriers exist for utilizing these treatments. Amid extensive studies and attempts to use CRISPR-Cas9 as an antivirus treatment for humans, problems such as effective method of delivery (Yip, 2020) and suppressing repair pathways for the damage (Liang et al., 2016) need to be solved for the CRISPR-Cas9 to be successfully used in human. Nonetheless, the technology of CRISPR Cas9 has shown to be effective in the treatment of infections such as HIV-1 (Xiao et al., 2019) and HBV (Kostyushev et al., 2019).

There are remarkable efforts to use CRISPR systems of bacteria and archaea for engineering DNA (Sorek et al., 2013) and RNA (Aman et al., 2018) virus interferences. Nonetheless, naturally occurring microRNAs have also shown to be effective in targeting and deactivating viruses. Zhen et al. showed that *Lonicera japonica* (Honeysuckle) contains atypical microRNA2911. This Chinese herb is used for treating influenza infection and the authors have provided evidence to show that this microRNA directly targets influenza virus and is the active ingredient of honeysuckle herb for the treatment of flu (Zhou et al., 2015). The effect of microRNA2911 on inhibiting varicella-zoster has also been reported (Huang et al., 2019). Here, we provide some evidence to support a hypothesis that using a prokaryote—Eukaryote symbiont might be a plausible training zone for producing therapeutic RNAs. The idea is when two living organisms constitute a symbiont in which one organism lacks defense mechanisms for viral infections, the whole symbiont fends off the pathogen. In this instant, a shared immune system is involved in which the adaptive immune elements can be used for both organisms.

*Dictyostelium discoideum* or social amoeba is a single cell eukaryote with a life cycle reminiscent to that of a non-equilibrium thermodynamic system; *D. discoideum* is a phagocytic cell which feeds mainly from soil bacteria and when facing starvation, starts aggregating and going through a multicellular development cycle (Molmeret et al., 2005). This amoeba has been subject to extensive genetic studies for good reasons: it phagocytoses bacteria (Molmeret et al., 2005) and large size viruses (Raoult and Boyer, 2010). This innate immune mechanism is, in all likelihood, responsible for the development of bacterial pathogenicity (Steinert and Heuner, 2005). This bacteria-amoeba interaction has made *D. discoideum* a melting pot for evolution and creation of new genetic materials (Raoult and Boyer, 2010). The plethora of lateral gene transfer in this melting pot has resulted in both amoeba (Eichinger et al., 2005) and the bacteria (Moliner et al., 2010) to have large genomes. By virtue of being host for several pathogens, *D. discoideum* is rich of noncoding RNAs (ncRNAs). The exact biological manifestation of many of these ncRNAs are yet to be discovered (Eddy, 2001). Although the life cycle of *D. discoideum* is invariant, its genetic material might change in each life cycle without expressing new proteins.

## New Approach

Here, we propose the possibility of using this Prokaryote-Eukaryote endosymbiosis for generating chimeric genetic material which can be used for suppressing RNA viruses. If the bacteria or the amoeba gets infected by a virus, a combination of Prokaryote-Eukaryote immune system will generate mechanism to protect the whole symbiont. This would be made possible through exchanging genetic material which can inhibit the virus. ncRNAs are perfect candidates for this proposed mechanism. The interaction between virus-microbiome-amoeba might provide us with naturally occurring genetic tools for inhibiting the expression of DNA or RNA viruses. In this cycle of interactions and the melting pot of genetic material, several possible genetic structures such as ncRNAs, antisense RNAs which can cause RNA silencing, might be produced. Finding a novel Ribonuclease (RNase) is another possibility.

This suggestion that *D. discoideum* needs to go through cycles of multicellular development is a speculation that during the multicellular phase in which cyclic AMP signals, some novel ncRNAs might develop. The behavior of prestalk and prespore cells are different and novel genetic material might be eliminated in the cycle through sloughing off of the stalk cells. In summary *D. discoideum* is a promising model to examine how the symbiont fends off a novel pathogen (mainly a virus) for which the amoeba does not have a well-established defense mechanism.

## Discussion

The efforts of using CRISPR-Cas9 technology for human gene editing and treatment of viruses is opening a new horizon for novel treatment methods. However, still there are several steps to take before we can safely and effectively use this technology for a viral infection treatment. Here I suggested a theoretical framework of using symbiotic relation between prokaryotes and eukaryotes for generating RNA-based therapeutics which might be used in human as well. This approach entails exposing the symbiont to a bacteriophage and searching for new genetic materials which are produced to eliminate the infection in the whole symbiont.

## Author Contributions

The author confirms being the sole contributor of this work and has approved it for publication.

## Conflict of Interest

The author declares that the research was conducted in the absence of any commercial or financial relationships that could be construed as a potential conflict of interest.
